# A regret theory approach to decision curve analysis: A novel method for eliciting decision makers' preferences and decision-making

**DOI:** 10.1186/1472-6947-10-51

**Published:** 2010-09-16

**Authors:** Athanasios Tsalatsanis, Iztok Hozo, Andrew Vickers, Benjamin Djulbegovic

**Affiliations:** 1 Center for Evidence-based Medicine and Health Outcomes Research, University of South Florida, Tampa, FL, USA; 2 Department of Mathematics, Indiana University Northwest, Gary, IN, USA; 3 Department of Epidemiology and Biostatistics, Memorial Sloan-Kettering Cancer Center, NY, NY, USA; 4 H. Lee Moffitt Cancer Center& Research Institute, Tampa, FL, USA

## Abstract

**Background:**

Decision curve analysis (DCA) has been proposed as an alternative method for evaluation of diagnostic tests, prediction models, and molecular markers. However, DCA is based on expected utility theory, which has been routinely violated by decision makers. Decision-making is governed by intuition (system 1), and analytical, deliberative process (system 2), thus, rational decision-making should reflect both formal principles of rationality and intuition about good decisions. We use the cognitive emotion of regret to serve as a link between systems 1 and 2 and to reformulate DCA.

**Methods:**

First, we analysed a classic decision tree describing three decision alternatives: treat, do not treat, and treat or no treat based on a predictive model. We then computed the expected regret for each of these alternatives as the difference between the utility of the action taken and the utility of the action that, in retrospect, should have been taken. For any pair of strategies, we measure the difference in net expected regret. Finally, we employ the concept of acceptable regret to identify the circumstances under which a potentially wrong strategy is tolerable to a decision-maker.

**Results:**

We developed a novel dual visual analog scale to describe the relationship between regret associated with "omissions" (e.g. failure to treat) vs. "commissions" (e.g. treating unnecessary) and decision maker's preferences as expressed in terms of threshold probability. We then proved that the Net Expected Regret Difference, first presented in this paper, is equivalent to net benefits as described in the original DCA. Based on the concept of acceptable regret we identified the circumstances under which a decision maker tolerates a potentially wrong decision and expressed it in terms of probability of disease.

**Conclusions:**

We present a novel method for eliciting decision maker's preferences and an alternative derivation of DCA based on regret theory. Our approach may be intuitively more appealing to a decision-maker, particularly in those clinical situations when the best management option is the one associated with the least amount of regret (e.g. diagnosis and treatment of advanced cancer, etc).

## Background

Decision making is often governed by uncertainty that inevitably affects the overall decision process. In their efforts to model uncertainty, decision theorists have proposed many methodologies with the majority of them having been based on statistics and probability[1-4], information theory and entropy[5], or possibilistic approaches such as fuzzy logic[6,7].

In clinical medical research, much effort has been invested in developing decision support systems for diagnosis and treatment of various clinical conditions such as management of infectious diseases in an intensive care unit, chronic prostatitis, or liver surgery[8-12]to name a few examples. Most of these systems are based on probabilistic prediction models. Even though prediction models have been shown to be generally superior and potentially complementary to physicians' prognostications [13-15], historically they have not fulfilled decision makers expectations to help improve decision-making. One reason for this is that most probabilistic medical decision support systems are based on expected utility theory that humans often violate[14,16,17]. In addition, most models in medicine do not incorporate decision-makers' preferences, which in addition to having reliable evidence, is the key to rational decision-making[18-20].

The goal of this paper is to develop a novel decision-making approach that incorporates the decision maker's attitudes towards multiple treatment strategies. Our goal is addressed through the following three specific aims. First, we deviate from the traditional expected utility theory in an attempt to satisfy both formal criteria of rationality and human intuition about good decisions[18-22]. We employ regret theory, since regret is a cognitive emotion that combines both rationality and intuition, which are key elements for decision-making[22,23], to develop a novel methodology for eliciting decision makers' personal preferences. Consequently we reformulate decision curve analysis (DCA)[24,25] from the regret theory point of view to evaluate alternative treatment strategies and to integrate both evidence on prognosis and treatment with the decision maker's attitudes and preferences[26-28]. Finally, we identify circumstances under which a decision maker tolerates a wrong decision.

To implement our approach, we first compute the threshold probability at which the decision maker is indifferent between alternative actions, based on the level of regret one might feel when he/she makes a wrong decision. We then employ the regret based DCA to identify the optimal strategy for a particular decision maker. The optimal strategy is the one that brings the least regret in the case that it is, in retrospect, wrong. We also show how to employ a prediction model to estimate the probability of disease for a patient and contrast it with the decision maker's threshold probability. Finally, we incorporate the concept of acceptable regret in the decision process to identify the conditions under which the decision maker tolerates a potentially wrong decision.

## Methods

### Decision analysis based on regret theory

**Figure 1:**
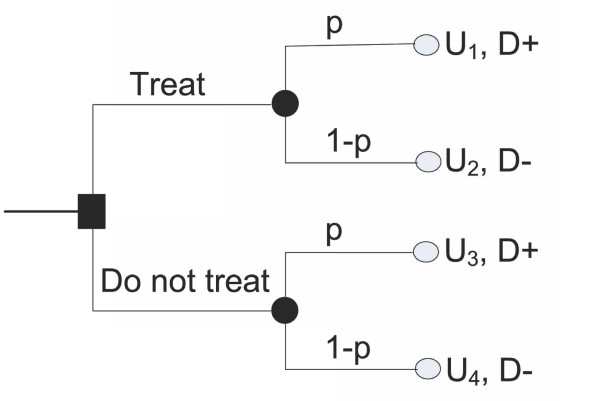
**Decision tree for administration of treatment**. In this figure, *p *= *P*(*D *+) is the probability associated with the presence of a disease; 1 *- p = P*(*D -*) is the probability associated with the absence of the disease; *U*_*i*_, i ∈ [Bibr B1][1,4], are the utilities corresponding to each outcome. Note that we use the term "treatment" in the generic sense of health care intervention, which may indicate therapy, procedure, or a diagnostic test.

Figure 1 depicts a typical decision tree describing administration of treatment guided by a prediction model. There are two competing strategies (treat, and do not treat), and four possible outcomes as described by the combinations: treat/do not treat and necessary/unnecessary.

In Figure 1, *p *= *P*(*D *+)is the probability associated with the presence of the disease as estimated by a prediction model;1 - *p *= *P*(*D *-)is the probability associated with the absence of the disease, and, *U*_*i*_, *i *∈ [1,4], are the utilities corresponding to each outcome. For example, *U*_1 _is the utility of administering treatment to a patient who has the disease (e.g. treat when necessary), and *U*_2 _is the utility of administering treatment to a patient who does not have the disease (e.g. administering unnecessary treatment). Note that we use the term "treatment" in the generic sense of health care intervention, which may indicate therapy, procedure, or a diagnostic test.

The probabilistic nature of prognostication models complicates significantly the decision process. For example, if a prediction model estimates the probability of a patient having a disease equal to 40%, it is unclear whether this patient should receive treatment or not. A solution from the point of view of the classical decision theory is to employ the concept of threshold probability *P*_*t *_, which is defined as the probability at which the decision maker is indifferent between two strategies (e.g. administer treatment or not)[27,29,30]. Based on the threshold concept, the patient should be treated if *p *≥ *P*_*t *_and should not be treated otherwise.

However, since in most cases decisions are made under uncertainty and can never be 100% accurate [23,26,28,31-34]. Thus, after a decision has been made one may discover that another alternative would have been preferable. This knowledge may bring a sense of loss or regret to the decision maker[23,26,28,31-34]. Regret can be particularly strong when the consequences of wrong decisions are life threatening or seriously influence the quality of the patient's life.

Formally, regret can be expressed as the difference between the utility of the outcome of the action taken and the utility of the outcome of the action that, in retrospect, should have been taken [23,26,28,31-34]. Regret can be felt by any party involved in the decision-making process (e.g. patients receiving treatment, patient's proxies or physicians administering treatment). For the rest of this paper we assume that the decision maker is the treating physician.

We first employ regret theory to estimate the threshold probability, *P*_*t *_, at which the physician is indifferent between alternative management strategies (e.g. administer treatment or not). In order to accomplish this, we describe regret in terms of the errors of (1) not treating the patient who has the disease, and (2) treating the patient who does not have the disease.

**Figure 2:**
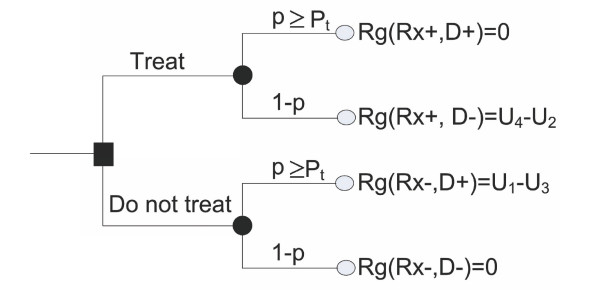
**Regret model of the decision tree for administration of treatment**. In this figure, *p*: probability of having the disease; 1-*p*: probability of not having the disease; *P*_*t*_: threshold probability for treatment; *Rg*: regret associated with wrong decisions; *Rx-*: no treatment; *Rx+*: treatment; *D+*: disease is present; *D-*: disease is absent. For example, *Rg*(*Rx+*, *D-*): regret associated with the error of treating the patient who did not have the disease.

Figure 2 describes the derivation of regret associated with each strategy based on the utilities of each action's outcome. As can be noted, the regret associated with the error of not treating the patient when he/she should have received treatment (the probability of disease is p ≥ *P*_*t*_), *Rg*(*Rx-*, *D*+), is equal to the loss in benefits of treatment. This can be expressed as the difference between the utility of receiving treatment and having the disease, and the utility of not receiving treatment and having the disease (U1-U3).

Similarly, the regret associated with treating the patient who should not have received treatment (the probability of disease is *p *<*P*_*t*_), *Rg*(*Rx*+, *D*-), is equal to harms incurred due to treatment. This can be expressed as the difference between the utilities of not having the disease and not receiving treatment, and not having the disease and receiving treatment (U4-U2). We expect no regret in the cases of correct treat/no treat decisions, *Rg*(*Rx*+, *D*+) = *Rg*(*Rx*-, *D*-) = 0. The difference (U1-U3) represents the consequences of not administering treatment where indicated, while (U4-U2) represents the consequences of administering treatment to a patient who does not need it. Under these assumptions, the threshold probability, *P*_*t *_is equal to [27,29,30]:

(1) $$P_{t}=\frac{1}{1+\frac{U_{1}-U_{3}}{U_{4}-U_{2}}}$$

Equation 1 effectively captures the preferences of the decision maker towards administering or not administering treatment. At the individual level, equation 1 shows how the threshold probability relates to the way the decision maker weighs false negative (i.e. failing to provide necessary treatment) vs. false positive (i.e. administering unnecessary treatment) results[24,25].

Note that the fraction $\frac{U_{1}-U_{3}}{U_{4}-U_{2}}$ is undefined for *U*_4 _- *U*_2 _= 0, which means that in this situation there is no regret associated with administering unnecessary treatment. Under these circumstances, *P*_*t *_= 100%, indicating that treatment is justified only in case of absolute certainty of disease (p = 100%), a realistically unachievable goal[26].

### Elicitation of threshold probability

There are numerous techniques for eliciting the decision maker's preferences regarding treatment administration [35]. None of them has been proven to be better than the other. We argue that any attempt to measure people's preferences and risk attitudes should be derived from an underlying theory of decision-making that can be applied to a problem or a class of the problems at hand. We approach elicitation of preferences by capturing people attitudes (e.g. physicians') through threshold probabilities. Normatively, a threshold probability reflects indifference between two alternative management strategies.

There are few commonly used methods to assess the value of this indifference for a decision maker such as the standard gamble, and the time trade-off [35-37]. The problem is that both standard gamble and time trade-off are time-consuming, cognitively more complex and are shown that can lead to biased estimates of people's preferences [36,37]. An alternative method is to use rating scales, such as visual analog scales (VAS), which are considerably easier to administer and better understood by the participants. The problem with analog scales, however, is that they cannot capture health state trade-offs[36,37].

The proposed method retains the simplicity of VAS but it takes into account the consequences of possible mistakes in decision-making by utilizing two visual analog scales. The first scale aims to assess the regret associated with potential error of failing to administer beneficial treatment ("regret of omission"). The second scale measures the regret of administration of unnecessary treatment ("regret of commission"). Using these two scales we can capture trade-offs and compute the threshold probability at which a decision maker is indifferent between two alternative management strategies.

We employed the two visual analog scales with typical 100 points [35-37]anchored by no regret and maximal regret. This is modeled after pain assessment limiting the maximum possible pain that a person can experience [38]. Accordingly, we can elicit threshold probabilities by asking the physician to weigh the regret associated with wrong decisions (e.g. giving unnecessary treatment vs. failure to administer necessary treatment) using a numerical (0 to 100) scale. The questions may be narrowly defined related to specific outcomes (e.g., survival/mortality, heart attack etc.). We should, however, note that most treatments are associated with multiple dimensions, some good and some bad. This is a fundamental reason why no universally accepted method for assessment of decision-makers' preferences has been developed so far. It is very difficult, if not impossible, to accurately determine the trade-offs across multiple outcomes that can be permuted in a number of ways. A solution to this problem is to capture the decision-maker's global or "holistic" perception toward treatment. By asking questions about trade-offs in this way, we directly address both cognitive mechanisms-intuitive and deliberative- of the decision process. This, in turn, can lead to more accurate assessment of the decision makers' preferences.

For example, to elicit the physician's threshold probability, we may ask the following questions:

*1. On a scale 0 to 100, where 0 indicates no regret and 100 indicates the maximum regret you could feel, how would you rate the level of your regret if you failed to provide necessary treatment to your patient (i.e. did not give treatment that, in retrospect, you should have given)? *[Note that the answer to this question corresponds to the (U1-U3) expression in equation 1)].

*2. On a scale 0 to 100, where 0 indicates no regret and 100 indicates the maximum regret you could feel, how would you rate the level of your regret if you had administered unnecessary treatment to your patient (i.e. administered treatment that, in retrospect, should have not been given)? *[Note the answer to this question corresponds to the (U4-U2) expression in equation 1).]

For example, suppose that the physician answers 60 and 30 to the questions 1 and 2, respectively. This means that the physician considers 60/30 = 2 times worse to fail to administer treatment that should have been given than to continue unnecessary treatment. Then, the threshold probability for this physician is:

$$P_{t}=\frac{1}{1+\frac{U_{1}-U_{3}}{U_{4}-U_{2}}}=\frac{1}{3}=33\%.$$

Thus, the physician would be unsure as to whether to treat or not the patient if the patient's probability of disease as computed by the prediction model was 33%. Thus, the recommended action, which is based on elicitation of the decision-maker preferences, is directly derived from the underlying theoretical model.

### Regret based decision curve analysis (DCA)

Decision-makers may be presented with many alternative strategies that can be difficult to model. A simple, yet powerful approach that is based on experience of a typical practicing physician is to compare the strategy based on modeling with those scenarios when all or no patient is treated. That is, the clinical alternatives to the prediction model strategy is to assume that all patients have the disease and thus treat them all, or to assume that no patient has the disease and thus treat none[25]. In this case the clinical dilemma a physician faces when considering treatment is threefold: (1) treat all the patients ("treat all"), (2)treat no patients ("treat none"), and (3) use a prediction model and treat a patient if *p *≥ *P*_*t *_("model").

The optimal decision depends on the preferences of the decision maker as captured by the threshold probability. We use Decision Curve Analysis (DCA) [24,25] to identify the range of threshold probabilities at which each strategy ("treat all", "treat none", and "model") is of value. Traditional DCA uses the (net expected) benefits associated with each strategy to recommend the best strategy [24,25]. In this work, we consider that the optimal strategy is the one that brings the least regret in case it is proven wrong, retrospectively.

One view about decision curves is that they should not be used in clinical practice: the researcher determines whether the decision curve justifies the use of the model in practice and then makes a simple recommendation yes or no as to whether clinicians should base their decisions on the model [39]. Another approach, which we propose here, is that threshold probabilities obtained in clinical practice should be compared against the decision curve to determine which strategy should be used (e.g. use a model, biopsy all men, biopsy no-one). This might be necessary if there is no strategy with the highest net benefit across the entire range of reasonable threshold probabilities.

**Figure 3:**
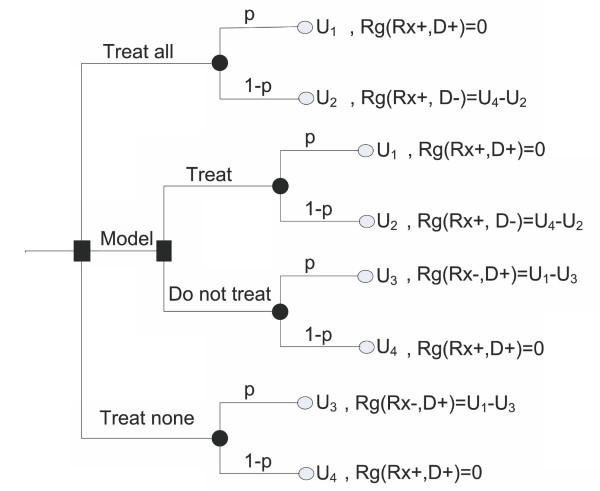
**Generalized decision tree for administration of treatment**. In this figure, *p*: probability of having the disease; 1-*p*: probability of not having the disease; *Rg*: regret associated with wrong decisions; *Rx-*: no treatment; *Rx+*: treatment; *D+*: disease is present; *D-*: disease is absent.

Figure 3, depicts the generalized decision tree describing all of the alternative strategies. By solving the decision tree, we can estimate the expected regret associated with each strategy[23,26,28,31-34]. For example,

(2) $$ERg[Model]=p(FN)(U_{1}-U_{3})+(1-p)(FP)(U_{4}-U_{2})$$

Here, *FN *(probability of false negatives) represents the conditional probability *P*(*p *<*P*_*t*_|*D *+)of not treating the patient who has the disease.

*FP *(probability of false positives) is the conditional probability *P*(*p *≥ *P*_*t*_|*D *-)of treating the patient who does not have the disease.

Similarly,

*TP *= 1 - *FN *= *P*(*p *≥ *P*_t_|*D *+)(probability of true positives): Probability of treating the patient who has the disease.

*TN *= 1 - *FP *= *P*(*p *<*P*_t_|*D *-) (probability of true negatives): Probability of not treating the patient who does not have the disease.

After re-scaling the utilities by dividing each utility with the expression *U*_1 _- *U*_3_, and replacing $\frac{U_{4}-U_{2}}{U_{1}-U_{3}}=\frac{P_{t}}{1-P_{t}}$, we get the expression:

(3) ERg[Model]=p(FN)+(1−p)(FP)Pt1−Pt=  p(1−TP)+(1−p)(FP)Pt1−Pt=P(p <Pt∩D+)+P(p≥Pt∩D−)Pt1−Pt

For the strategies of administering treatment and not administering treatment, the expected regret is derived as:

(4) $$ERg[Treat\;all]=(1-p)(U_{4}-U_{2})=(1-p)\frac{P_{t}}{1-P_{t}}$$

(5) $$ERg[Treat\;none]=p(U_{1}-U_{3})=p$$

Subtracting each of these expected regrets from the expected regret of the "Treat none" (baseline) strategy we obtain the "**Net Expected Regret Difference (NERD)**":

(6) NERD [Treat none, Model]=ERg[Treat none]−ERg[Model]==p−p(1−TP)−(1−p)(FP)Pt1−Pt=p(TP)−(1−p) (FP)Pt1−Pt

(7) NERD [Treat none, Treat al]=ERg[Treat none]−ERg[ Treat all]=p−(1−p)Pt1−Pt

(8) NERD[Treat none,Treat none]=0

Note that these are **exactly **the same formulas as those derived by Vickers and Elkin [25] who employ the expected-utility model in "decision curve analysis" (DCA). The regret based derivation, however, is mathematically more parsimonious. The original DCA formulation required several mathematical manipulations making the simplicity of regret approach more attractive. In addition, as argued throughout the manuscript, the regret formulation may have additional decision-theoretical advantages as it enables experiencing consequences of decisions both at the emotional (system 1) and cognitive (system 2) level[23,40].

In addition to equations 6-8, we are interested in the NERD between the strategies "Treat all" and "Model":

(9) $$\begin{array}{l} NERD[Treat\;all,\;Model]=ERg[Treat\;all]-ERg[Model]\\ =(1-p)(TN)\frac{P_{t}}{1-P_{t}}-p(FN) \end{array}$$

The NERD equations associated with each strategy, 6-8, can be further reformulated as follows [23,25,26,28,31-34,41]:

(10) NERD=P(p≥Pt∩D +)−P(p≥Pt∩D −)Pt1−Pt=#TPn−#FPn⋅Pt1−Pt

Similarly, equation 9 can be re-written as:

(11) NERD=(1−p)(TN)Pt1−Pt−p(FN)=P(p <Pt∩D −)Pt1−Pt−P(p <Pt∩D +) =#TNn⋅Pt1−Pt−#FNn

Equations 10 and 11 above are useful when calculating *NERD *as a function of *P*_*t*_. The probabilities *P*(*p *≥ *P*_*t *_∩ *D *+), *P*(*p *≥ *P*_*t *_∩ *D *-), *P*(*p *≥ *P*_*t *_∩ *D *+), and *P*(*p *≥ *P*_*t *_∩ *D *-) are estimated as follows:

• *P*(*p *≥ *P*_*t *_∩ *D *+) ≈ the number of patients who have the disease and for whom the prognostic probability is greater than or equal to *P*_*t*_(with #TP = number of patients with true positive results, $P\left(p\geq P_{t}\cap D+\right)\approx \frac{\#TP}{n}$, where *n *is the total number of patients in the study).

• *P*(*p *≥ *P*_*t *_∩ *D *-) ≈ the number of patients who do not have the disease and for whom the prognostic probability of disease is greater than or equal to *P*_*t *_(with #FP = number of patients with false positive results, $P\left(p\geq P_{t}\cap D–\right)\approx \frac{\#FP}{n})$.

• *P*(*p *<*P*_*t *_∩ *D *+) ≈ the number of patients who have the disease and for whom the prognostic probability of disease is less than *P*_*t *_(with #TN = number of patients with true negative results, $P\left(p<P_{t}\cap D+\right)\approx \frac{\#TN}{n})$.

• *P*(*p *<*P*_*t *_∩ *D *-) ≈ the number of patients who do not have the disease and for whom the prognostic probability of disease is less than *P*_*t *_(with #FN=number of patients with false negative results, $P\left(p<P_{t}\cap D–\right)\approx \frac{\#FN}{n})$.

When computing *NERD*[*Treat none, treat all*] we assume that all patients have the disease, thus #TP is the number of people who actually have the disease and #FP is the number of people who do not have the disease but are given treatment. On the other hand, when computing *NERD*[*Treat none, Model*]from equation 10 and, *NERD*[*Treat all, Model*]from equation 11, #TP, #FP, #TN, and #FN are computed for each threshold probability assuming that a patient has the disease if the prognostic probability is greater than or equal to the threshold probability and does not have the disease, otherwise.

NERDs of each of the strategies described are plotted against different values of threshold probability. The NERD values provide information relative to **decrease in regret **when two strategies are compared against each other for a given threshold probability. If NERD = 0, this means that there is no difference in the regret between two strategies:

(12) $$\begin{array}{c} NERD[strategy\;1,\;strategy\;2]=0\Leftrightarrow \\ ERg(strategy1)-ERg(strategy2)=0\Leftrightarrow \\ ERg(strategy1)=ERg(strategy2) \end{array}$$

If NERD > 0, this means that the second strategy will inflict less regret than the first strategy, and hence it is preferable:

(13) $$\begin{array}{c} NERD[strategy\;1,\;strategy\;2]>0\Leftrightarrow \\ ERg(strategy1)>ERg(strategy2) \end{array}$$

Similarly, if NERD < 0, the first strategy represents the optimal decision among the two strategies:

(14) $$\begin{array}{l} NERD[strategy\;1,\;strategy\;2]<0\Leftrightarrow \\ ERg(strategy1)<ERg(strategy2) \end{array}$$

The algorithm for the Regret DCA is implemented as follows:

1. Select a value for threshold probability.

2. Assuming that patients should be treated if *p *≥ *P*_*t *_and should not be treated otherwise, compute #TP and #FP for the prediction model.

3. Calculate the *NERD*(*Treat none, Model*)using equation 10.

4. Calculate *NERD*(*Treat all, Model*)using equation 11.

5. Compute the *NERD*(*Treat none, Treat all*)using equation 10 where #TP is the number of patients having the disease and #FP is the number of patients without disease who got treatment.

6. Repeat steps 1 - 6 for a range of threshold probabilities.

7. Graph each NERD calculated in steps 3-5 against each threshold probability.

Based on the Regret DCA methodology, the optimal decision at each threshold probability is derived by comparing each pair of strategies through their corresponding NERDs according to the transitivity principle (i.e., if A > B, B > C then A > C). Thus, if *NERD*(*strategy*1, *strategy*2) >*NERD*(*strategy*2, *strategy*3) > 0 then strategy 2 is better than strategy 1, and strategy 3 is better than strategy 2. Therefore, strategy 3 is the optimal strategy.

### Acceptable Regret

No decision model can guarantee that the recommended strategy will be the correct one. Therefore, we can always make a mistake and recommend treatment we should not have, or fail to recommend treatment we should have administered [42]. However, there are situations where the regret resulting from a wrong decision will be tolerable. These situations are best described under the notion of acceptable regret [26,28,31]. Formally, acceptable regret,*Rg*_0_, is defined as the portion of utility a decision maker is willing to lose/sacrifice when he/she adheres to a decision that may prove wrong [26,28,31,32]. For example, a physician may regret administering unnecessary treatment to a patient but he/she can "still live with" the consequences of this decision if she/he judged them to be trivial or inconsequential.

We assume that there is a linear relationship between the value of acceptable regret and the benefits of receiving treatment as well as the harms of receiving unnecessary treatment. This is a reasonable assumption because acceptable regret is expected to operate within a narrow range, at the lower or the upper end, of the probability scale. We define acceptable regret in terms of benefits of treatment,*Rg_b_*, as [43] the percentage (*r_b_*) of benefits (*U*_1 _- *U*_3_)the decision maker is willing to forgo if his/her decision NOT to treat was wrong:

(15) $$Rg_{0}=Rg_{b}=r_{b}B=r_{b}(U_{1}-U_{3})$$

Alternatively, we define acceptable regret in terms of harms of unnecessary treatment, *Rg*_*h*_, as[43] the percentage (*r*_*h*_) of harms (U_4 _- U_2_) the decision maker is willing to incur if his/her decision of treating was wrong:

(16) $$Rg_{0}=Rg_{h}=r_{h}H=r_{h}(U_{4}-U_{2})$$

We use the concept of acceptable regret to further refine the conditions under which the decision maker is indifferent between two strategies. Recall that these conditions have been initially captured in terms of threshold probability, which does not incorporate the sense of tolerable losses. Thus, we proceed with the following definition: Two strategies are considered *equivalent in regret *(e.g. will bring the same regret to the decision maker if they are proven wrong, in retrospect), if the absolute value of their net expected regret difference (NERD) is less than or equal to a predetermined amount of acceptable regret *Rg*_0_. In other words, there is no difference between choosing the strategy "treat all" or "treat none" in terms of regret if:

(17) $$|NERD\;(Treat\;none,Treat\;all)|\;\leq Rg_{0}$$

Similarly, the strategies "model" and "treat none" are equivalent in regret if:

(18) $$NERD(Treat\;none,Model)|\;\leq Rg_{0}$$

and the strategies "model" and "treat all":

(19) $$|NERD(Treat\;all,Model)|\;\leq Rg_{0}$$

The acceptable regret,*Rg*_0_, can be computed using any of the two definitions described in equations 15 and 16.

We can also use equations 15 and 16 to identify the prognostic probabilities at which the decision maker would not regret the decision to which he/she is committed even if that decision may prove wrong. For instance, we are typically interested in the prognostic probability above which a physician would commit to the decision to treat a patient, and the probability below which he/she would not to treat a patient without feeling undue consequences of these decisions[28]. In other words, we are looking for the probabilities for which *ERg*(*Treat all*) ≤ *Rg*_*h*_, and *ERg*(*Treat none*) ≤ *Rg*_*b*_. Solving the inequalities using equations 4, 5, 15, and 16 and after scaling *Rg*_*0 *_by (*U*_1 _- *U*_3_), we obtain

(20) $$P_{treat\;all}=1-r_{h}$$

Where *P_treat all _is *the prognostic probability above which the physician would tolerate giving treatment that may prove unnecessary. Similarly,

(21) $$P_{treat\;none}=r_{b}$$

represents the prognostic probability below which the physician would comfortably withhold treatment that may prove beneficial, in retrospect.

Note that equations 20 and 21 express acceptable regret in terms of probabilities while equations 17-19 define it in terms of NERD. Hence, the outputs of these equations are not the same; rather, they complement each other.

### Elicitation of acceptable regret

In most cases the decision maker does not have a complete understanding of benefits lost or harms inflicted and cannot assign a precise number to them. For this reason, we do not suggest inquiring directly about the value of *r*. Instead, we propose eliciting *r *through the decision-maker's responses to specific clinical scenarios. For example, we propose the following approach:

*Assume that you have **100 patients with the same probability of disease as the patient you are currently treating**. You need to decide whether each of these patients should receive treatment or not. Since no prediction model is 100% accurate, it is expected that you will make some mistakes in your treatment recommendations (e.g. you may recommend treatment to a patient who does not need it, or fail to recommend treatment to a patient who needs it)*.

*1. We are now interested in knowing your tolerance toward administering **unnecessary **treatment i.e. we want to learn what the magnitude of the **unavoidable error **you can live with is by inflicting potentially harmful treatment on a patient. Note that if you say that your acceptable regret is zero, this means that you can only make decision if you **absolutely certain **that your recommendation is correct*.

*Out of the number (100-y) of patients who should have not received treatment, how many patients would you tolerate treating? *(The answer is used to compute r_*h*_).

*2. We are interested in knowing your tolerance toward **failing **to provide necessary treatment i.e. we want to learn what the magnitude of **unavoidable error **you can live with is by forgoing potentially beneficial treatment. Note that if you say that your acceptable regret is zero, this means that you can only make decision if you **absolutely certain **that your recommendation is correct*.

*Out of the number (100-x) of patients who should have been treated, how many patients would you tolerate not treating? *(The answer is used to compute r_*b*_).

It is unnecessary to ask the decision maker to answer both questions. We suggest asking only the question related to the recommendation the physician is about to make e.g. if the recommendation is about administering treatment, then the decision maker should be asked the second question, while if it is about not giving treatment, then he/she can ask the first question.

The value of acceptable regret is plotted in the regret DCA graph to visually facilitate the decision making process. At a specific threshold probability all strategies for which |*NERD*| ≤ *Rg*_0 _are considered equivalent in regret, according to the definition in the previous section.

## Example

We will employ a prostate cancer biopsy example to demonstrate the applicability of our approach. Prostate cancer biopsy is an invasive and uncomfortable procedure, which can be painful and is associated with a risk of infection. However, it is often necessary for diagnosis of prostate cancer, one of the leading causes of cancer death in men.

Men are typically biopsied for prostate cancer if they have an elevated level of prostate-specific antigen (PSA). However, most men with a high PSA do not have prostate cancer. This has led to the idea that statistical models based on multiple predictors (PSA, age, family history, other markers) might be used to predict biopsy outcomes and hence aid biopsy decisions for individual patients. A physician seeing a patient with an elevated PSA has three possible options: go for biopsy, refuse biopsy or look up his probability in a statistical model and then make a decision.

**Figure 4:**
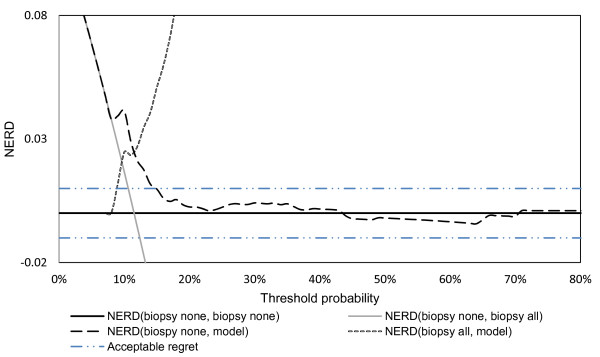
**Regret DCA regarding biopsy to detect prostate cancer**. Thin line: biopsy all patients; solid line: biopsy no patients; dashed line: prediction model. The optimal strategy is derived by the comparison of each pair of strategies from all NERDs as per equations 12-14. The statistical model is the optimal strategy for threshold probabilities between 8% and 42%. For threshold probabilities between 43% and 95%, the optimal strategy is to biopsy no patients, while for 0% to 8% both "model" and "biopsy all" strategies are optimal. The lines of acceptable regret denote the regret area in which different strategies are equivalent.For example, at threshold probability equal to 20%, the optimal strategy is acting based on the prognostic model. However, NERD(biopsy none, model) is below the acceptable regret line which indicates that the strategies "biopsy none" and "model" are equivalent in regret. Therefore the optimal strategy is to biopsy no patients as the use of model is deemed to be superfluous. Similarly,at threshold probability equal to 15%, the optimal strategy is to act based on the model and the strategies "biopsy all" and "biopsy none" are equivalent in regret. Finally, at threshold probability equal to 9%, the optimal strategies are both "model" and "biopsy all". However, since NERD(biopsy all, model) is below the acceptable regret, the strategies "biopsy all" and "model" are equivalent in regret. Therefore the optimal strategy is to biopsy all patients.

We utilize an unpublished statistical model that computes probability of cancer based on the dataset described in [44] to compare each of these options. Following the algorithm described in the regret DCA section, we generate the decision curves depicted in Figure 4. This figure is used to determine the optimal strategy for different values of threshold probability. The optimization procedure is implemented in three steps where the strategies in each NERD are compared to each other as in equations 12- 14 at a specific threshold probability. For example, at threshold probability 15%:

1. *NERD*(*biopsy none,model*) > 0 therefore, the model is preferred to the strategy biopsy none.

2. *NERD*(*biopsy none, biopsy all*) > 0 therefore, the strategy biopsy all is preferred to the strategy biopsy none.

3. *NERD*(*biopsy all,model*) > 0 therefore, the model is preferred to the strategy biopsy none

Consequently, "model" corresponds to the optimal strategy.

Repeating the same procedure for all threshold probabilities, we can see that deciding based on the statistical model is the optimal strategy (i.e. results in the minimum expected regret) for threshold probabilities between 8% and 43%. For threshold probabilities between 42% and 95%, the optimal strategy is to biopsy no patients, while for 0% to 8% both model and biopsy all strategies are optimal.

To interpret these results, we have to consider how a typical physician values the harms of a false negative (missing a cancer) and a false positive (an unnecessary biopsy) result. If regret associated with unnecessary biopsy is felt to be worse than missing cancer, then according to equation 1, the threshold probability is greater than 50%. However, it is unlikely that a physician would consider an unnecessary biopsy to be worse than missing a cancer, so the threshold probability for biopsy must be less than 50%. Thus, a reasonable range of threshold probabilities might indeed be between 8% - 43% as suggested by our model. As the model is superior across this entire range, we can conclude that, ***irrespective of the physician's exact preferences***, making a biopsy decision based on the statistical model will lead to lower expected regret than an alternative such as biopsying all or no men. Based on discussions with clinicians, we believe that a reasonable range of threshold probability is 10% - 40%. As the regret associated with the model strategy is lowest across this entire range, we can recommend use of the model. Nonetheless, we do not have a complete sample of all physician preferences and it is possible that a physician may have a probability outside of this range.

To illustrate the applicability of the acceptable regret model, assume that the value of acceptable regret for forgoing the benefits of biopsy (equation 15) is equal to ± 0.01. Consider the case that the decision maker's threshold probability is equal to 20%. According to Figure 4, the optimal strategy should be suggested by the statistical model. However, we see that

|NERD(treat none, model)| <0.01

which means that the strategies "biopsy none" (biopsy no patients) and "model" are equivalent in regret. Therefore, the prediction model does not offer any better information and thus, it can be disregarded.

## Case Study

This section describes the overall decision process regarding prostate cancer biopsy. The process begins with elicitation of the threshold probability from the treating physician and continues with evaluation of the available strategies based on regret DCA (Figure 4). Then, if necessary, the probability of cancer based on the available prognostic model is computed and contrasted with the threshold probability. Finally, the concept of acceptable regret is employed to arrive at the strategy which is the most tolerable to the decision maker who always faces possibilities of making wrong decisions. For the remainder of this section the normal font text corresponds to the author comments. The text in **bold **and underlined font corresponds to questions to, and answers from the physician respectively. The *italic *text is notes to the reader. We demonstrate the applicability of our approach using hypothetical answers from two physicians.

The overall decision process is described as follows:

1. Interview with the physician to elicit his/her threshold probability.

a. **On the scale 0 to 100, where 0 indicates no regret and 100 indicates the maximum regret you could feel, how would you rate your level of regret if you failed to provide necessary treatment?**

Physician #1 answer: 50, Physician #2 answer: 70. *These values correspond to U*_1 _- *U*_3 _*from equation1*.

b. **On the scale 0 to 100, where 0 indicates no regret and 100 indicates the maximum regret you could feel, how would you rate your level of regret if you administered unnecessary treatment?**

Physician #1: 10, Physician #2: 60. *This value corresponds to U*_4 _- *U*_2 _*from equation 1*.

*The threshold probability is equal to (equation 1): Physician #1: 16%, Physician #2: 46%*.

2. Using the graph in Figure 4, identify the optimal strategy for the computed threshold probability.

Physician #1: *For threshold probability equal to 16%, the optimal decision is derived by solving the inequalities *(Figure 4, *equations 12-14):*

1. NERD(biopy all, model) > 0, *the strategy "model" is better than the strategy "biopsy all"*

2. NERD(biopsy none, model) > 0, *the strategy "model" is better than the strategy "biopsy none"*

3. NERD(biopsy none, biopsy all) > 0, *the strategy "biopsy all" is better than "biopsy none"*.

*Therefore, the optimal strategy is the "model" which corresponds to biopsy based on the probability of cancer predicted by the statistical model. The next step is to compute the patient's probability of cancer and contrast it with the threshold probability*.

Physician #2: *For threshold probability equal to 46%, the optimal decision would be the "biopsy none" strategy. In this case, even though computing the probability of cancer will not affect the physician's decision, it will help identify the circumstances under which the physician would tolerate unnecessary biopsy of the patient*.

3. Compute the cancer probability for the specific patient based on the statistical model.

*a. If the cancer probability is greater than or equal to the threshold probability, then the surgeon should biopsy the patient*.

*b. If the cancer probability is less than the threshold probability, then the surgeon should not biopsy the patient*.

*Let us assume that the probability of cancer for the specific patient is equal to 20%. The threshold probability for Physician #1 is 16% (as computed in step 1). In this case, Physician #1 considers recommending biopsy. As noted in step 2b, the best strategy for Physician #2 is recommending not to biopsy any patients regardless their probability of cancer*.

4. Elicitation of the level of acceptable regret.

**Assume that you have 100 patients, all with probability of cancer equal to 20% (the same as your patient). This means that out of 100 patients, 20 patients will have cancer while 80 will not have cancer. You need to decide whether each of these patients should undergo biopsy or not. Since no prediction model is 100% accurate, it is expected that you will make some mistakes in your recommendations (e.g. you may recommend biopsy to a patient who does not need it, or fail to recommend biopsy to a patient who may need it)**.

*a. The physician considers biopsy *(Physician #1):

**Out of the 20 patients who should be biopsied, for how many patients would you tolerate not recommending a necessary biopsy? **1.

*This answer corresponds to *$r_{b}=\frac{1}{20}=0.05$*and acceptable regret Rg*_b _= *r_b_*(*U*_1 _- *U*_3_)*=*0.05 _* _0.5 = 0.025. *The optimal strategy at P*_*t *_= 16% *is to use the statistical model* (Figure 4). *For P*_*t *_= 16% *and Rg*_*b *_= 0.025 *all NERDs are greater than acceptable regret, thus the optimal strategy remains the statistical model*.

*b. The physician does not consider biopsy *(Physician #2).

**Out of the 80 patients who should not undergo biopsy, for how many patients would you tolerate recommending an unnecessary biopsy? **40.

*The answer provided by the Physician #2 corresponds to *$r_{h}=\frac{40}{80}=0.50$* and acceptable regret Rg*_*h *_= *r*_*h*_(*U*_4 _- *U*_2_) = 0.5 _* _0.6 = 0.3.

*The optimal strategy for P*_*t *_= 46% *is to biopsy no patients (Figure *4). *Also, for p*_*t *_= 46% *and Rg*_*h *_= 0.3, *we have: *|*NERD(biopsy none, biopsy all*)| = | -0.639 >*Rg*_*h*_, |*NERD(biopsy none, model*)| = | - 0.003| <*Rg_h _and *|*NERD(biopsy all, model*) = 0.6364 >*Rg*_*h*_. *This means that the strategies "biopsy none" and "model" are equivalent in regret. In practical terms no additional effort is justified for using the statistical model*.

5. Based on equations 20 and 21, we can determine the prognostic probabilities above and under which the physician would tolerate performing an unnecessary biopsy, or not to do so when he should have done it.

*a. Physician #1 considers recommending biopsy to his/her patient. Based on equation 21, the physician would tolerate not recommending a biopsy for any prognostic probability below P_treat none _*= *r_b_* = 5%.

*b. Physician #2 considers not recommending biopsy to his/her patient. Based on equation 20, the decision maker would tolerate recommending an unnecessary biopsy for any prognostic probability above P_treat all _*= 1 - *r_h _*= 50%

## Discussion

Currently, there is no agreed upon method for how preferences regarding multiple objectives that typically go in opposite directions (i.e. most medical interventions are associated both with benefits and harms) should be elicited. We have presented and demonstrated an approach to decision making based on regret theory and decision curve analysis. The approach presented in this paper relies on the concept of the threshold probability at which a decision maker is indifferent between strategies, to suggest the optimal decision [27,29,30]. Unlike the approaches described in the classic threshold papers [27,29,30], our approach is based on the notion that the value of threshold probability is clearly subjective and depends on the personal preferences of the decision maker. We elicit threshold probabilities based on the regret one may feel in case that the chosen strategy is proven wrong, in retrospect. Although one can narrow down the approach to specific medical outcomes, we believe that eliciting preferences in a global, holistic way is more useful if our approach is to be used in the actual practice.

We believe that the model described here has a direct practical application in overcoming many difficulties related to linking evidence with patient's preferences to arrive at the optimal decision- the issues that plagued the field of decision-making. The problem of eliciting preferences and integrating them in a coherent decision is not a simple one. We argue that the approach we are advocating here represents a contribution to the field of decision making, be should not be seen as the panacea to medical decision making. However, we anticipate our methodology to be suitable for medical decision primarily associated with trade-offs between quality and quantity of life.

Over that last couple of decades, many attempts have been made to develop the best method to take these considerations in real-life settings. Unfortunately, as explained, no approach has succeeded [35]. We believe that the reason for this is that most approaches to elicit decision maker's preferences as well as to help improve decision-making have relied on a rational framework based on expected utility theory[21]. However, modern cognitive theories (within so called dual-processing theory) have convincingly demonstrated that human decisions rely both on intuition (system 1) and analytical, deliberative process (system 2) in balancing risks and benefits in the decision-making process [22,40,45]. We believe that rational decision-making should take into account both formal principles of rationality and human intuition about good decisions [46,47]. The key is to preserve rational framework, while allowing anticipation of the effect of decision on emotions (while avoiding biases associated with intuitive thinking) [40]. One way to accomplish this is to use the cognitive emotion of regret to serve as a link between system 1 (i.e. intuitive system) and system 2 (i.e. deliberative, analytical cognitive system). By anticipating consequences of our actions and circumstances under which we can live with our mistakes, we bring together both aspects of cognition that may lead to better and more satisfactory decision-making.

Specifically, we argue that eliciting people's preferences using regret theory may be superior to using traditional utility theory because regret forces decision-makers to explicitly consider consequences of decisions. We have previously shown that we can always make errors in decision-making: recommend treatment that does not work, or fail to recommend treatment that does [26]. Therefore, we reformulated DCA from the regret theory's point of view. Furthermore, it has been shown that the expected utility theory is often violated to minimize anticipated regret [33,34].In addition, there is substantial evidence that medical decision making aims to minimize regret associated with wrong decisions [48-50].

Moreover, while descriptive, normative, and prescriptive theories [17] tend to evaluate individual outcomes, the approach presented here evaluates all of the outcomes in a holistic manner. Our approach is consistent with Reyna's "gist" or "fuzzy trace theory" in which the decision-maker characterizes gist of each outcome to arrive at a given decision [51]. For example, consider that a decision maker is provided with a list of harms and benefits associated with each decision, as it is currently recommended by the practice guidelines panels [52]. In traditional theories, the decision maker evaluates a treatment strategy by reasoning on each of the harms and benefits associated with a given strategy. This, as discussed above, would mean integration of all multiple outcomes that often go in different directions typically within limited time-frame. Due to the complexity of these decisions, however, this approach overwhelms the decision maker as our brain capacity is limited. The regret DCA methodology quantifies the global attitudes of the decision maker towards a specific strategy without requiring separate reasoning for each of the harms and benefits. This holistic assessment occurs within the dual processing cognitive system, which evaluates collectively the harms and the benefits associated with each treatment alternative. By assessing trade-offs through both cognitive mechanisms-intuitive and deliberative- we believe that we can assess decision makers' preferences more accurately.

In general, since our method relies on the elicitation of threshold probability we recommend using our methodology for every patient. As every patient's values are different the threshold probability should indeed be patient-specific. For example, a physician may act "aggressively" for a young patient who is the father of two underage kids and less aggressively for an older patient. However, in the cancer biopsy example, it is expected that most of the patients should present with similar characteristics and therefore most physicians would settle in a small area of threshold probabilities. In this case repeating the elicitation process for every patient would be impractical. Nevertheless, this is an empirical question worthy of further investigation as alluded above.

Our approach may help reconcile formal principles of rationality and human intuitions about good decisions that may better reflect "rationality" in medical decision-making [21,32,46,47]. We hope that our theoretical work will stimulate empirical testing of the concepts outlined in this paper. Toward this end, we are currently working on developing a prescriptive computerized decision-support system to facilitate the application of the model described herein. Such a system is expected to be user friendly with built-in automatic manipulation of the complex calculations that may be off-putting to many users. We hope to report on testing of our system in the near future.

## Conclusions

We have presented a decision making methodology that relies on regret theory and decision curve analysis to assist physicians in choosing between appropriate health care interventions. Our methodology utilizes the cognitive emotion of regret to determine the decision maker's preferences towards available strategies and DCA to suggest the optimal decision for the specific decision maker. We believe that our approach is suitable for those clinical situations when the best management option is the one associated with the least amount of regret (e.g. diagnosis and treatment of advanced cancer, etc).

As with any other novel theoretical work, our approach has its limitations. First, it has not been empirically tested in a clinical setting. However, we are in the process of developing the appropriate decision support tools to bring our model into clinical practice and evaluate its usefulness with actual physicians and patients. Second, the methodology presented is appropriate for single point decision making. Further investigation is required to determine the application of regret theory to decisions that re-occur over time. Finally, we assume that there is only one decision maker involved in the decision process. Nevertheless, our plan for future work includes extending our methodology to shared decision-making that will include both physician and patient in the decision process and investigate whether in practice there is a difference between preferences and choices made by physicians and their patients.

We summarize the contribution presented in this paper as follows:

1. We propose a novel method for eliciting decision makers' preferences towards treatment administration. Contrary to traditional methodologies on eliciting preferences, our method considers the consequences of potential mistakes in decisions. We propose a dual visual analog scale to capture errors of omission and errors of commission and, therefore, evaluate the trade-offs associated with each of the available strategies.

2. We have reformulated DCA from the regret theory point of view. Our approach is intuitively more appealing to a decision maker and should facilitate decision making particularly in those clinical situations when the best management option is the one associated with the least amount of regret.

3. Finally, we utilize the concept of acceptable regret to identify the circumstances under which a decision maker tolerates a wrong decision.

We envision facilitation of the decision process in clinical settings through a computerized decision support system available at the point of care. In fact, we are in the process of developing such a system and hope to report about it soon.

## Abbreviations

DCA: Decision Curve Analysis; NERD: Net Expected Regret Difference; VAS: Visual Analog Scale, *p*: Prognostic probability; *P_t_*: Threshold probability; *D *+/*D *-: The patient has/does not have the disease; *U_i_*: Utility corresponding to outcome *I*; *Rg*(*x*): Regret associated with the action *x*; *Rx *+/*Rx *-: Treatment/No treatment; *U*_1 _- *U*_3_: Consequences of not administering treatment where indicated; *U*_4 _- *U*_2: _Consequences of unnecessarily administering treatment; *ERg*(*action*): Expected regret associated with an action; *TP,TN,FP,FN*: Conditional probabilities; #*TP*,#*TN*,#*FP*,#*FN*: Number of *TP*, *TN*, *FB*, *FN *patients; *n*: Number of patients; *NERD*(*action*1, *action*2): Net expected regret difference between actions 1 and 2; *Rg*_0_: Acceptable regret; *Rg_b_*: Acceptable regret as defined in terms of loses in benefits due to forgoing treatment; *Rg_h_*: Acceptable regret as defined in terms of harms due to undergoing unnecessary treatment; *r_b_*/*r_h_*: Percentages of the benefits/harms a decision maker is willing to lose/incur in case of a wrong decision; *P_treat all _*: The prognostic probability above which the decision maker would tolerate recommending unnecessary treatment; *P_treat none_*: The prognostic probability below which the decision maker would tolerate not recommending treatment.

## Competing interests

The authors declare that they have no competing interests.

## Authors' contributions

AT prepared the first draft, formalized the proposed methodology, and applied it into treatment administration examples; IH developed the mathematical formulation of the model; AV is the author of DCA; BD proposed the regret theory extension to DCA. All authors contributed equally in reviewing multiple versions of the paper and provided important feedback to the final version of the paper. BD is a guarantor. All authors read and approved the final draft.

## Pre-publication history

The pre-publication history for this paper can be accessed here:

http://www.biomedcentral.com/1472-6947/10/51/prepub
